# mRNA vaccine development during the COVID-19 pandemic: a retrospective review from the perspective of the Swiss affiliate of a global biopharmaceutical company

**DOI:** 10.1186/s40545-023-00652-y

**Published:** 2023-11-27

**Authors:** Tim Killeen, Vanessa Kermer, Rahel Troxler Saxer

**Affiliations:** 1 Medical Affairs, Pfizer AG, Schärenmoosstrasse 99, 8052 Zurich, Switzerland; 2 Regulatory Affairs, Pfizer AG, Schärenmoosstrasse 99, 8052 Zurich, Switzerland; 3 Postgraduate Training Centre for Pharmaceutical Medicine, Pfizer AG, Schärenmoosstrasse 99, 8052 Zurich, Switzerland

**Keywords:** Coronavirus disease 2019, Messenger ribonucleic acid, Vaccines, Regulatory, Clinical development, Safety, Supply, Switzerland, Pandemic, Public health

## Abstract

The coronavirus disease 2019 (COVID-19) pandemic has been the defining public health emergency of our time. In Switzerland, messenger RNA (mRNA) vaccines were and still are widely utilized as a critical component of the Federal Office of Public Health (FOPH)’s preventative mitigation strategy. The development, conditional approval and worldwide roll-out of mRNA vaccines against COVID-19 proceeded at an unprecedented pace and presented myriad challenges for manufacturers. In this review, we discuss, from the perspective of the Swiss affiliate of a global biopharmaceutical company, the clinical, regulatory, pharmacovigilance and logistical considerations of making a mRNA COVID-19 vaccine available to the Swiss population during a pandemic as rapidly as possible while ensuring strict adherence to safety and quality standards.

## Methodology

In this narrative review, we aimed to collate the available information relating to COVID-19 mRNA vaccine development and roll-out in the specific Swiss regulatory and pandemic setting, complemented by our observations from the perspective of one of the pharmaceutical companies developing and manufacturing such a vaccine. We performed internet and literature searches focusing on the period from early 2020 to early 2021, during which the pivotal studies were conducted, and first conditional approvals were granted in Switzerland.

## Background

COVID-19 is a highly transmissible respiratory infection caused by the severe acute respiratory syndrome coronavirus 2 (SARS-CoV-2). Emergent in Wuhan, China in December 2019, it was declared a pandemic on 11th March 2020 [1]. Although most patients experience a mild-to-moderate course, severe disease leads to significant morbidity and mortality, with > 57 000 hospitalizations and > 13 500 deaths recorded in Switzerland to September 2022 [2]. In addition to this health burden, the COVID-19 pandemic necessitated unprecedented restrictions on travel, social, educational and economic activity [3].

The SARS-CoV-2 genome was sequenced within weeks of the initial outbreak in Wuhan [4]. Like other coronaviruses, SARS-CoV-2 comprises four structural proteins, one of which, the spike surface (S) glycoprotein, is responsible for binding to the host cell—in the case of SARS-CoV-2 mainly via the angiotensin-converting enzyme 2 receptor (ACE2) [5]. Knowledge of the S protein structure presented a potential therapeutic target for vaccines based on mRNA [6, 7].

## Clinical development

**Fig. 1:**
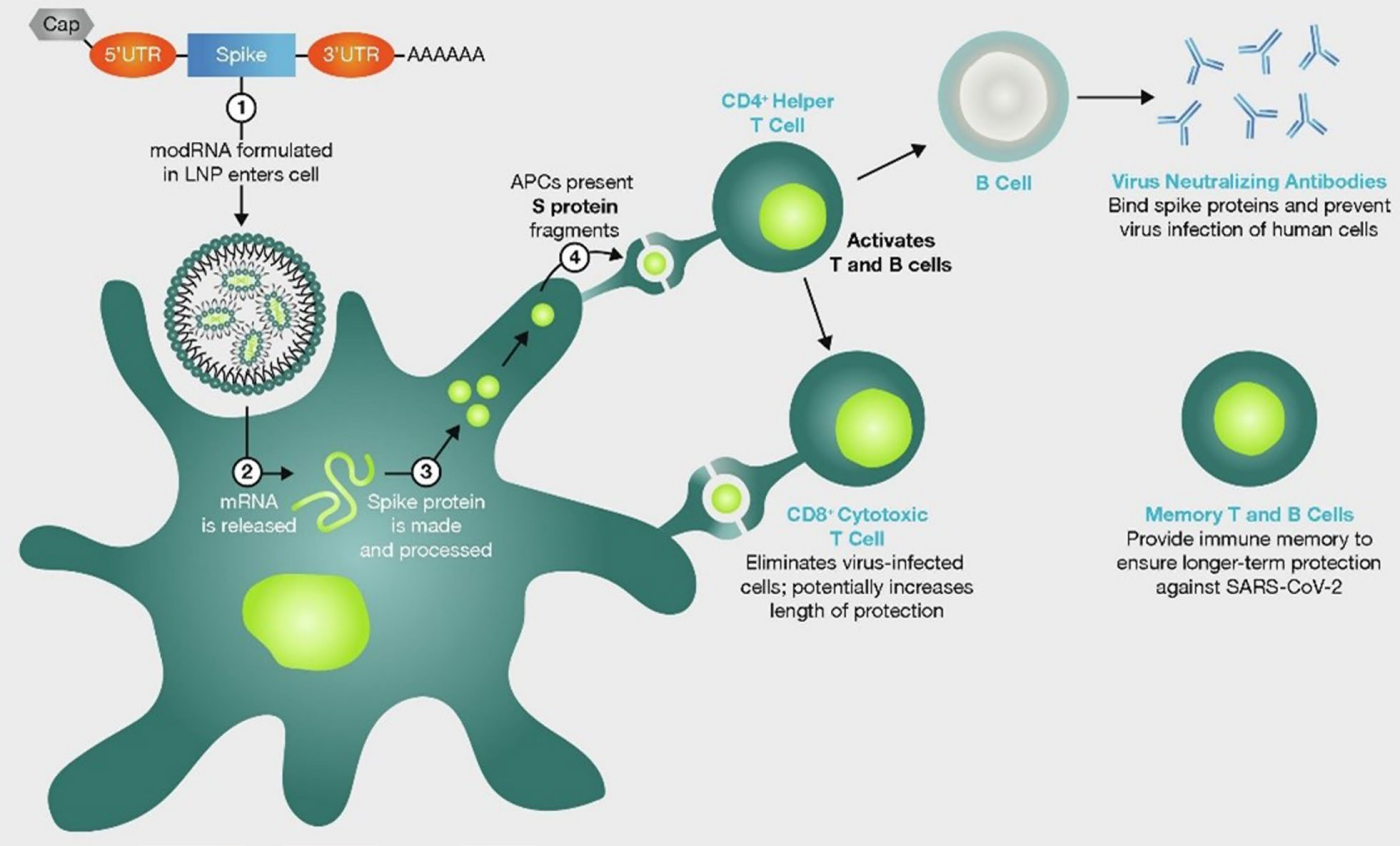
mRNA COVID-19 vaccines mechanism of action. Simplified graphical representation of the mode of action of mRNA vaccines against COVID-19. AAAAAA; poly-A tail of adenine nucleotides, APC; antigen presenting cells, CD; cluster of differentiation, LNP; lipid nanoparticles, mRNA; messenger ribonucleic acid, modRNA; modified ribonucleic acid, S; spike, SARS-CoV-19; severe acute respiratory syndrome coronovirus 2019, UTR; untranslated region. Image reproduced from Thomas SJ et al., Vaccine 2022;40(10):1483–92 (https://doi.org/10.1016/j.vaccine.2021.12.046) under Creative Commons CC-BY-NC-ND public licence available at https://creativecommons.org/licenses/by-nc-nd/4.0/

Under investigation since the 1990s, mRNA vaccines involve the delivery of mRNA encoding a given protein into the recipient’s cells, where the target protein is subsequently produced, eliciting an immune response (Fig. 1) [6, 8]. mRNA vaccines encoding the SARS-CoV-2 include mRNA-1273 (Spikevax®; Moderna) and BNT162b2 (Comirnaty®; BioNTech/Pfizer) [1, 9]. Both vaccines utilize similar innovations for successful clinical translation via intramuscular injection into the deltoid. These include the envelopment of the payload mRNA in a lipid nanoparticle, modification to stabilize the resultant S protein in the configuration prior to which SARS-CoV-2 fuses with ACE2 receptors, and the replacement of uridine residues with pseudouridine to enhance translation and inhibit potential immune responses to the mRNA itself [1, 6–9].

**Fig. 2:**
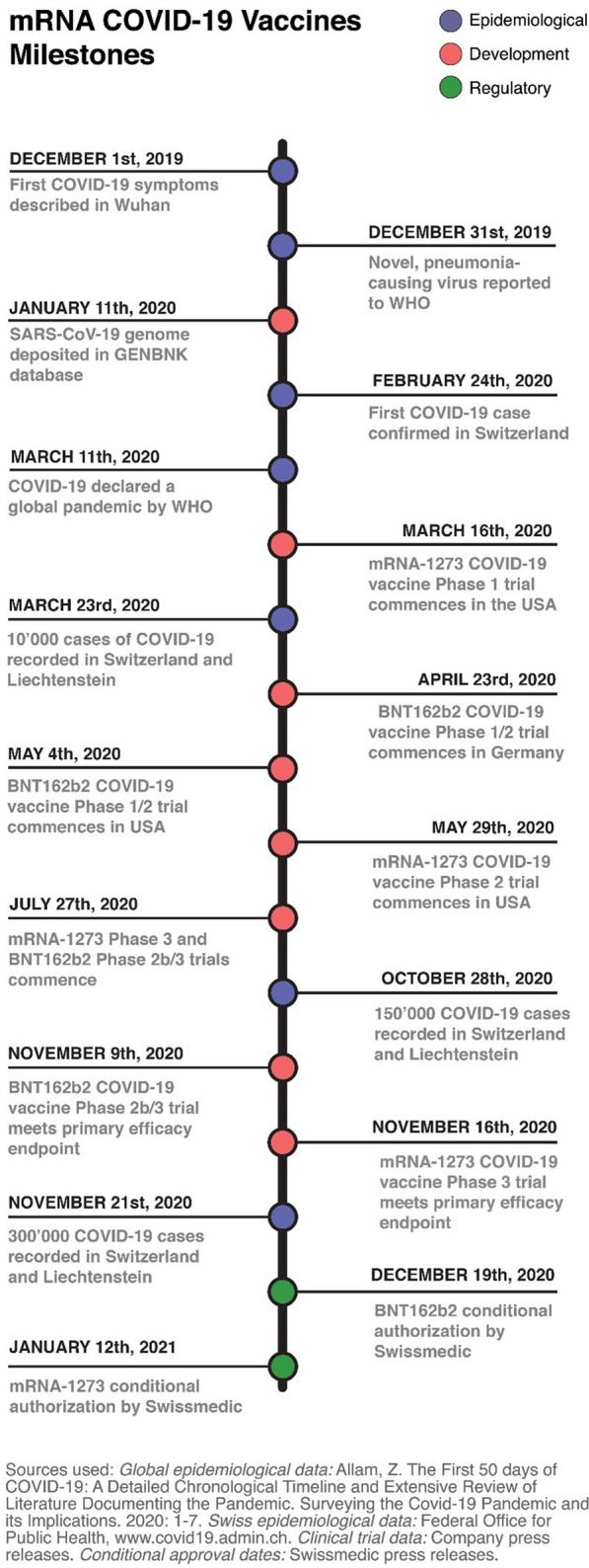
mRNA COVID-19 vaccines milestones. Timeline showing selected epidemiological, clinical and regulatory events during the first year of the COVID-19 pandemic with an emphasis on Switzerland. WHO; World Health Organization

In light of the severity of the pandemic threat, both mRNA-1273 and BNT162b2 underwent unprecedentedly accelerated clinical development (Fig. 2), with both vaccines requiring only 11 months from program initiation to first emergency use approvals [7]. Early phase results were promising [10, 11] and both programs proceeded to large-scale pivotal trials, which met their primary efficacy endpoints in December 2020 [1, 9]. In a phase 2/3, placebo-controlled, observer-blinded trial of BNT162b2 (NCT04368728), 43 548 volunteers aged 16 and over received two doses of either placebo or 30µg BNT162b2 21 days apart. The vaccine was 95% (credible interval 90.3–97.6%) efficacious at preventing COVID-19 7 days after the second dose [1]. Similarly, in a phase 3 randomized, observer-blinded, placebo-controlled trial (NCT04368728) including 30 420 adult volunteers who received two doses of either placebo or 100μg mRNA-1273 28 days apart, the vaccine exhibited 94.1% (confidence interval 89.3–96.8%) efficacy in preventing COVID-19 at least 14 days after the second dose [9]. The safety profile of these mRNA vaccines in these trials was characterized by short-term, mild-to-moderate injection site pain, fatigue, headache, chills, myalgia and arthralgia, and serious adverse events were rare [1, 9].

A meta-analysis of Phase II/III randomised, controlled trials of mRNA vaccines against COVID-19 conducted using data up to March 2021 reported efficacy of 94.6% (95% CI 0.936–0.954), robust immune responses and adverse event profiles in line with those reported in the individual pivotal studies for mRNA-1273 and BNT162b2 outlined above [12].

## Regulatory review of BNT162b2 (Comirnaty®)

The usual route to marketing authorization of a medicinal product with a new active substance in Switzerland is to submit an application as set out in Article 11 of the therapeutic products act (TPA) [13, 14], before which a complete dossier, including read-outs of clinical trials and details on validated manufacturing processes, must be prepared. A standard timeline of 540 calendar days from submission is foreseen for the authorization of a new therapeutic product. On 18th September 2020, based on the COVID-19 ordinance 3 article 21, the Swiss medicines regulator Swissmedic clarified significantly faster, alternative routes for medicinal products targeting COVID-19, utilizing novel “rolling review” concepts [15, 16]. In a regular review, the applicant must submit all relevant data at formalized milestones, at which point activity switches to Swissmedic, who review the dossier and issue a consolidated list of questions to which the submitting company responds within a certain timeframe. Rolling review does away with this structure—the company submits data and dossier components as they become available and Swissmedic responds with queries as they arise [16]. In addition, TPA Article 9a [13] allows Swissmedic to temporarily authorize medicinal products, provided that a series of preconditions are met regarding the severity of the disease in question and anticipated effectiveness of the medicinal product and assumes that the company will provide the required data to convert the authorization into an ordinary authorization before the temporary authorization expires [16].

While the aforementioned pivotal trial was still ongoing, and on the strength of the Phase 1/2 data, Pfizer AG submitted BNT162b2 into a rolling submission process with Swissmedic in mid-October 2020, which was placed on a track towards temporary authorisation via Article 9b by Swissmedic [13, 14, 16]. As the Phase 3 data became available in December, an intense period of correspondence between Pfizer’s regulatory affairs department and Swissmedic ensued, with both parties working long and irregular hours. At this time, Switzerland was recording more than 600 COVID-19-related deaths per week[3], and as in other Swiss businesses, the vast majority of Pfizer AG staff were working from home, further complicating this exceptional workload. On 19^th^ December, 64 days after the submission of the first data was submitted, Swissmedic granted temporary authorisation to Comirnaty® for active immunisation in persons aged 16 and over for prevention of COVID-19. This represented the world’s first approval of a COVID-19 vaccine outside of emergency use programs [15].

A few weeks later, on 12th January 2021, mRNA-1273 (Spikevax®) was similarly granted temporary authorisation for use in those aged 18 and over for protection against COVID-19 [17].

This process of rolling review, while undoubtably key to the unprecedentedly rapid timelines achieved for mRNA vaccines in the pandemic setting, presented significant challenges to the usual internal processes established by pharmaceutical companies and regulatory authorities. Workforce and resource planning was complicated by timelines dictated primarily by the availability of data, ultimately tied to event occurrence in clinical trials.

## Supply and logistics

The manufacture of an mRNA vaccine consists of a production step, in which the mRNA is produced from a DNA template via in vitro transcription, and a downstream phase during which the mRNA is purified to remove undesired mRNA species and non-mRNA proteins and encapsulated in lipid nanoparticles [7, 8]. For a comprehensive review of mRNA vaccine manufacture beyond the scope of this article, see Rosa et al. [8]. Despite this relatively simple process (compared to most conventional vaccines) [7], the pressing nature of the public health emergency meant that manufacturing was required at unprecedented scale and speed. At-risk manufacturing development was scaled-up much earlier in clinical development than would normally be the case, without deviation from Good Manufacturing Practice (GMP) standards [7]. Nevertheless, supply in late 2020/early 2021 was very limited [2], and vaccines were initially restricted to people at particular risk for poor outcomes [18].

Under the epidemic law/COVID-19 ordinance, many aspects of procurement and supply of vaccines were exceptionally undertaken centrally by the FOPH. For reasons of thermostability, Comirnaty® and Spikevax® require very low temperatures (− 90 to − 60°C and − 50 to − 15°C, respectively) for storage and/or transport [19]. For Comirnaty, this required the coordination of an end-to-end, ultra-low temperature (ULT) cold chain from the factory in Puurs, Belgium to vaccination centres in Switzerland. This was achieved by shipping the vaccine vials in trays secured within thermal containers cooled with dry ice. This unusual requirement necessitated training of physicians and pharmacists in the safe handling of dry ice and the support of the Swiss military pharmacy for logistics and distribution. Instructional materials were prepared and continuously updated in the national languages and educational training was provided to cantonal stakeholders by colleagues in our medical department.

## Drug safety and risk management in a rapidly changing environment

**Fig. 3:**
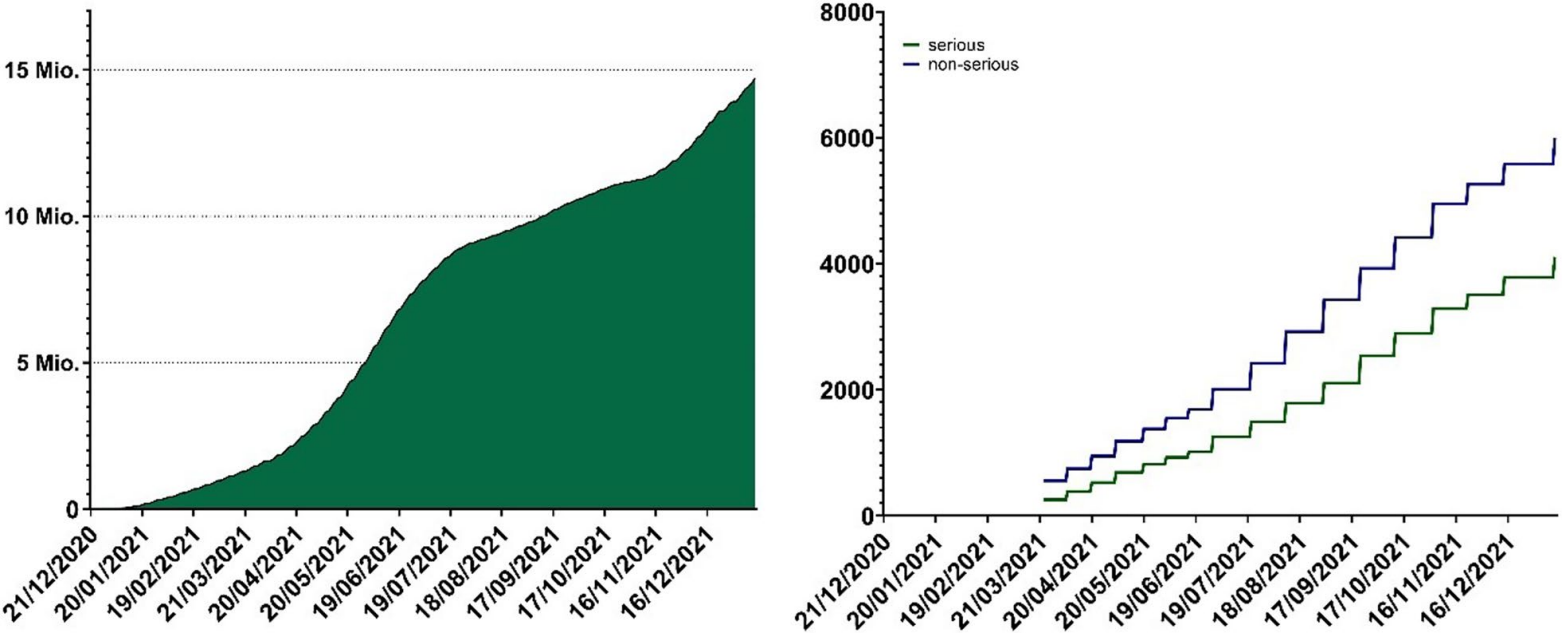
Vaccine administration and adverse vaccination reaction reporting in Switzerland and Liechtenstein. Left: cumulative total administered doses of mRNA vaccines from the start of the Swiss vaccination program to the end of 2021 (mRNA-1273 and BNT162b2 were the only conditionally approved mRNA COVID-19 vaccines during this perioxd). Federal Office of Public Health, www.covid19.admin.ch. Right: cumulative totals of adverse vaccination reactions (blue; non-serious, green; serious) following mRNA vaccine administration reported to Swissmedic during the same period. Data on adverse vaccination reactions were generally released weekly during this period. Source: Federal Office of Public Health https://www.covid19.admin.ch/api/data/20220705-0r3tf4ch/sources/COVID19VaccSymptoms.csv. Accessed 15 Nov 2023

For most of 2021, mRNA vaccines were the only type of COVID-19 vaccine available in Switzerland. During this period, just over 13.9 million doses of mRNA COVID-19 vaccines were given in Switzerland with approximately 8.8m doses of Spikevax and approximately 5m doses of Comirnaty administered [2]. Comprehensive pharmacovigilance mechanisms were put in place by Swissmedic and internationally to monitor adverse events and detect emergent safety signals. As vaccination use increased in Switzerland and internationally, pharmacovigilance professionals in the companies and in regulatory authorities absorbed and acted upon unprecedentedly high volumes of safety reports (Fig. 3), which were carefully reviewed and reported via established pharmacovigilance pathways.

As a result of this scrutiny, important safety information emerged in the months after the conditional approval of mRNA vaccines. Allergic reactions to the vaccination, including very rare (defined by regulators as < 1/10,000) cases of anaphylaxis, were observed and precautionary management steps outlined in Swiss guidelines for this important identified risk [18, 19]. This included the requirement for vaccinees to remain at the vaccination centre and be observed for at least 15 min after vaccination [18]. Subsequently, a safety signal was confirmed for the very rare occurrence of myocarditis and pericarditis within 14 days of vaccination, more commonly after the second dose and in younger males [19]. In consultation with Swissmedic, a letter (“Dear Health Care Professional letter”) was sent to health care professionals in Switzerland by Pfizer and Moderna communicating this and providing relevant advice [20].

## Ongoing evolution

As the pandemic evolved and more clinical data became available through ongoing clinical trials and post-approval surveillance, an improved understanding of the benefit/risk profile of mRNA COVID-19 vaccines has seen indications extended to younger populations and updates in formulations and storage temperature requirements. The Comirnaty prescribing information underwent 12 approved revisions in 2021 alone, ensuring that the most current scientific data were available to Swiss healthcare professionals.

We encourage all readers to familiarise themselves with the product information for all COVID-19 vaccines, which can be found at www.swissmedicinfo.ch. In addition, a summary of Risk Management Plans (RMP) summaries can be accessed at: https://www.swissmedic.ch/swissmedic/en/home/humanarzneimittel/market-surveillance/risk-management--psurs--pv-planning-/rmp-summaries.html

## Limitations

This article lays out the chronology and important unique aspects of mRNA vaccine development, regulatory review, approval and roll-out from our perspective—that of one pharmaceutical company. While we attempted to review and accurately present all the information in the public domain related to mRNA-1273, our insights are necessarily more limited for certain aspects, particularly regulatory interactions and supply considerations, for which no public record exists.

## Conclusions

The COVID-19 pandemic profoundly touched the lives of people of all walks of life across Switzerland, personally and professionally. The availability of mRNA COVID-19 vaccines was a major positive milestone in the pandemic and, along with other measures, has saved millions of lives[21], relieved pressure on the healthcare system and allowed a gradual return to normality. In this review, we highlighted the unprecedented scientific, regulatory, logistical and pharmacovigilance challenges associated with making mRNA vaccinations available in Switzerland. The innovation and learning engendered through this process continue to be applied in the COVID-19 pandemic and leave us better prepared for future public health emergencies.

## Data Availability

Data supporting this review was taken from public repositories and sources are given in the legends of the respective figures.

## Abbreviations

ACE2: Angiotensin converting enzyme 2
COVID-19: Coronavirus disease 2019
FOPH: Swiss Federal Office of Public Health
GMP: Good Manufacturing Practice
mRNA: Messenger ribonucleic acid
SARS-CoV-2: Severe acute respiratory syndrome coronavirus 2
TPA: Therapeutic Products Act
ULT: Ultra low temperature
