# Correction: mRNA vaccine development during the COVID-19 pandemic: a retrospective review from the perspective of the Swiss affiliate of a global biopharmaceutical company

**DOI:** 10.1186/s40545-023-00677-3

**Published:** 2023-12-11

**Authors:** Tim Killeen, Vanessa Kermer, Rahel Troxler Saxer

**Affiliations:** 1 Medical Afairs, Pfzer AG, Schärenmoosstrasse 99, 8052 Zurich, Switzerland; 2 Regulatory Afairs, Pfzer AG, Schärenmoosstrasse 99, 8052 Zurich, Switzerland; 3 Postgraduate Training Centre for Pharmaceutical Medicine, Pfzer AG, Schärenmoosstrasse 99, 8052 Zurich, Switzerland

**Correction** **:** **Journal of Pharmaceutical Policy and Practice (2023) 16:158** 10.1186/s40545-023-00652-y

Following publication of the original article [1], it was reported that there were two incorrect references to “liquid nitrogen” in the ‘Supply and logistics’ section. These two instances have been replaced with “dry ice” as follows (correction in bold):

- This was achieved by shipping the vaccine vials in trays secured within thermal containers cooled with **dry ice**. This unusual requirement necessitated training of physicians and pharmacists in the safe handling of **dry ice** and the support of the Swiss military pharmacy for logistics and distribution.

The original article has been updated.
